# Pediatric Traffic Injuries on Halloween in the United Kingdom: Prevalence and Injury Severity

**DOI:** 10.3390/ijerph18179093

**Published:** 2021-08-28

**Authors:** Li-Min Hsu, Bayu Satria Wiratama, Ping-Ling Chen, Wafaa Saleh, Hui-An Lin, Chih-Wei Pai

**Affiliations:** 1 Graduate Institute of Injury Prevention and Control, College of Public Health, Taipei Medical University, Taipei 110, Taiwan; slm09002@yahoo.com.tw (L.-M.H.); d513107004@tmu.edu.tw (B.S.W.); plchen@tmu.edu.tw (P.-L.C.); m513108004@tmu.edu.tw (H.-A.L.); 2 Department of Surgery and Traumatology, National Taiwan University Hospital, Taipei 100, Taiwan; 3 Department of Epidemiology, Biostatistics and Population Health, Faculty of Medicine, Public Health and Nursing, Universitas Gadjah Mada, Yogyakarta City 55281, Indonesia; 4 Transport Research Institute, Edinburgh Napier University, Scotland EH11 4DY, UK; w.saleh@napier.ac.uk; 5 Department of Emergency Medicine, Taipei Medical University Hospital, Taipei 110, Taiwan

**Keywords:** Halloween, pediatric traffic injury, injury severity

## Abstract

The study results serve as a reminder for parents, children, and drivers to be alert to the danger of traffic crashes on Halloween. The aim of this study was to examine whether Halloween is associated with a higher incidence of traffic injuries and whether traffic injuries sustained on Halloween are more severe than those sustained on other days. The U.K. STATS19 database, including the data of all road traffic crashes occurring from 1990 to 2017, was employed. A total of 73,587 pediatric traffic casualties (involving pedestrians, cyclists, and moped riders) were included. Between 17:00 and 19:00 (17:00~18:59) on Halloween, the number of casualties was higher than that on other public holidays and usual days. The logistic regression model revealed that, between 17:00 and 18:00 (17:00~17:59), the risk of being killed or seriously injured on Halloween was 34.2% higher (odds ratio = 1.342; 95% CI = 1.065–1.692) than that on other days. Pediatric crashes occurring on Halloween are associated with a higher number of injuries and increased injury severity.

## 1. Introduction

Injury is a leading cause of death in children [1]. Pedestrian injury is the second leading cause of injury-related death in the United States for children aged 5 to 14 years [2]. Almost one-fifth (19%) of children aged 14 years and younger who were killed in traffic crashes were found to be pedestrians [3]. Children living in an urban or high-density area are at higher risk of being injured or killed as a pedestrian. Incidents resulting in child pedestrian fatalities are the most frequent during spring and fall, with the highest number of child pedestrian deaths caused by motor vehicle crashes occurring during May and October [4].

On holidays, many activities are held in which people participate in a creative environment where injuries commonly occur. The holidays with the highest number of injuries per year were discovered to be Labor Day, Memorial Day, the Fourth of July, and Halloween [5]. Researchers have evaluated the injury risk associated with specific holidays. Some studies have investigated the occurrence of firework-related injuries on the Fourth of July [6,7], whereas other studies have determined the risk of hand lacerations during the carving of Halloween pumpkins [8]. Research focusing on Christmas has investigated eye injuries caused by Christmas trees [9], inhalation of Christmas ornaments, ingestion of decorations, and effects of toxic plants such as mistletoe [10].

On average, 41 million children trick-or-treat every Halloween. In the day and in the evening on this specific holiday, children are outdoors trick-or-treating, walking along sidewalks and roadways, and crossing streets. Children are particularly vulnerable to injuries as a pedestrian. Because their cognitive, developmental, behavioral, physical, and sensory abilities are not as efficient as those of adults, they tend to have difficulty making appropriate judgment when faced with a traffic threat. Parents often misestimate their child’s ability to judge pedestrian danger, which makes this phenomenon even more concerning [11]. Children are impulsive and have not yet developed the skills to judge how far away a car is and how quickly it is approaching [12].

Both children and parents must understand the potential dangers that children face on Halloween and the steps they can take to remain safe. In this study, we proposed two research hypotheses: first, more traffic injuries occur on Halloween than on other days of the year; and second, traffic injuries sustained on Halloween are generally more severe than those sustained on other days of the year.

## 2. Materials and Methods

### 2.1. Study Design and Setting

The current research used the U.K. STATS19 database, which contains the data of all road traffic accidents. Crashes that result in personal injury must be reported to the police within 30 days. The variables collected in this study were crash, vehicle, and casualty characteristics. This study was approved by the Taipei Medical University Joint Institutional Review Board (N202011030. MOST 110-2410-H-038-016-MY2).

### 2.2. Casualties

This study focused on child casualties who were pedestrians and bicyclists involved in crashes with a bicycle, motorcycle, or motor vehicle. On Halloween, celebrations such as trick-or-treating generally begin at dusk (i.e., after 16:00); thus, data were collected for the period of 16:00 to 00:00 (16:00~23:59). Pediatric casualties aged 4 to 17 years were included in the analysis. We excluded casualties with missing data for sex, age, speed limit, crash time, or vehicle type. A total of 73,587 casualties over the period from 1990 to 2017 were included in the final dataset.

### 2.3. Outcome and Variable Definitions

In the STATS19 database, the injury severity levels are fatal, serious, and slight injuries. The definition of serious injury is as follows: injury that results in hospitalization or any of the following injuries: fracture, concussion, internal injuries, crushing, and severe general shock requiring medical treatment. Slight injuries include mild injuries such as sprains (including whiplash), bruises, and cuts requiring roadside attention. Injuries not requiring medical treatment are also classified as slight injuries. Fatal injury is defined as injuries that cause death fewer than 30 days after the accident. Fatal and serious injuries are combined together as killed or seriously injured (KSIs).

Types of vehicles involved in pediatric crashes include bicycles, motorcycles, automobiles, and large vehicles. Weather was categorized as either fine or adverse, which included rain and snow. Road surface condition was categorized as dry and not dry. U.K. car speed limits are generally 30 mph in urban areas, 60 mph on main single-carriageway roads, and 70 mph on dual carriageways and motorways. Thus, we used 30 mph as a threshold for urban and rural road type. Roads with speed limits of 30 miles per hour and higher were defined as rural, whereas those with speed limits lower than 30 miles per hour were defined as urban.

Days on which crashes occurred were classified into four categories: Halloween (31 October), public holidays, weekdays, and weekend days. The public holidays considered were New Year’s Day, Good Friday, Easter Monday, summer bank holidays, spring bank holidays, Christmas Day, Boxing Day, the Queen’s 2002 Golden Jubilee, and the Queen’s 2012 Diamond Jubilee.

Because the activities on Halloween start in the evening and last until midnight, during this period, the children may be particularly vulnerable to traffic injuries during their activities. Hourly variation in injury risk was examined, with a focus on crashes that occurred between 16:00 and 00:00 (16:00~23:59). Injury prevalence (for both all injuries and KSIs) and injury severity (the proportion of KSIs) were evaluated.

### 2.4. Statistical Analysis

The primary analysis compared the mean hourly numbers of casualties and KSIs across the four categories of days. In addition, to investigate the effect of Halloween on injury severity, we compared the proportion of KSIs among casualties across sex, weather condition, road type, vehicle type, and day type subgroups in each period using the chi-square test. A multiple logistic regression model was used to explore the associations between potential risk factors and KSIs and to calculate adjusted odds ratios (AORs). An alpha value of 0.05 was used, yielding a confidence level of 95%. Complete case analysis was performed in this study. Missing data were considered to be at least missing at random, and cases with missing data were thus excluded from the analysis.

## 3. Results

Table 1 shows the characteristics of casualties that occurred from 1990 to 2017. A total of 73,587 casualties were included in this study; more casualties occurred among girls than among boys (59.28% vs. 40.72%). As high as 91% of crashes occurred in an urban setting. More than 90% of casualties resulted from a crash with an automobile. Among these casualties, almost 24.77% were classified as KSI. The number of casualties was higher on weekdays, which accounted for 71.16% of the total, followed by public holidays (approximately 20.80%), Halloweens (1.91%), and weekend days (1.13%).

**Figure 1:**
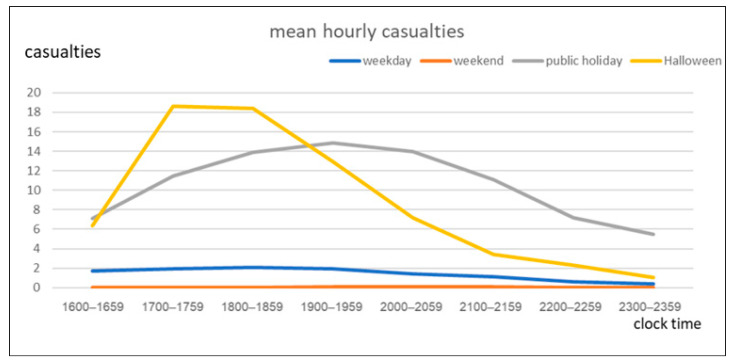
Mean hourly casualties by type of day.

**Figure 2:**
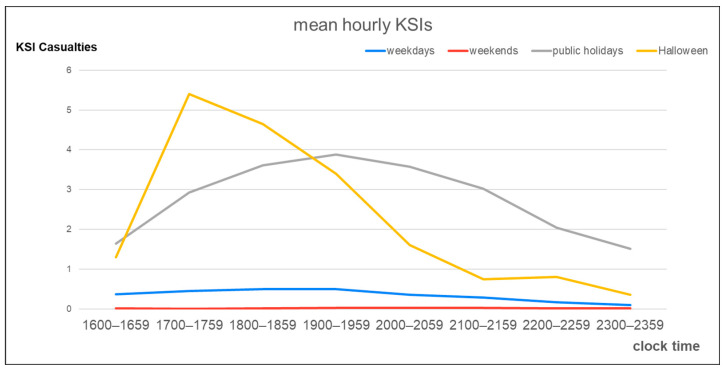
Mean number of hourly KSIs by type of day.

Figure 1 presents the mean number of hourly casualties over the period from 16:00 to 00:00 (16:00~23:59). From 17:00 to 19:00 (17:00~18:59), the number of casualties was higher on Halloweens (18.6 and 18.4 cases/h, respectively) than on the other day types. Figure 2 illustrates the mean number of hourly KSIs for the different day types. During the hours from 17:00 to 19:00 (17:00~18:59), the number of KSIs per hour was the highest on Halloweens (5.4 and 4.6 cases/h, respectively).

Table 2 presents the distribution of KSIs by each independent variable. Boys were more likely to sustain KSIs than girls (25.87% vs. 23.17%). The KSI risk was higher on rural roads (40.73%) than on urban roads (23.22%). The KSI percentage was the highest for children involved in a crash with a large vehicle (30.65%) compared with crashes with the other vehicle types. In the period from 17:00 to 18:00 (17:00~17:59), the proportion of KSIs showed a significant difference, which was the highest for crashes on Halloweens (28.95%) compared with crashes on other day types.

Table 3 presents the results of logistic regression analysis of KSI risk factors. From 17:00 to 16:00 (17:00~17:59), pediatric casualties involved in a crash on Halloween were 34.2% more likely to sustain KSIs than were those involved in a crash on a different day type (AOR = 1.342; 95% CI = 1.065–1.692). Other KSI risk factors included a crash involving an automobile (AOR = 1.443; 95% CI = 1.108–1.879), when the casualties were boys (AOR = 1.156; 95% CI = 1.117–1.197), a crash occurring in a rural area (AOR = 2.261; 95% CI = 2.144–2.385), fine weather (AOR = 1.209; 95% CI = 1.151–1.274), and a wet road surface (AOR = 1.1; 95% CI = 1.051–1.143).

## 4. Discussion

Our research indicates that, between 17:00 and 19:00 (17:00~18:59) on Halloween, the numbers of all injuries and specifically KSIs among children injured in a traffic crashes were higher than those on all the other days and holidays. In the event that crashes occurred between 17:00 and 18:00 (17:00~17:59) on Halloween, pediatric casualties were 34% more likely to sustain KSIs than those that occurred on a different day and in different periods. Two points relating to our results are worthy of note: (a) both all injuries and KSIs are more prevalent between 17:00 and 19:00 (17:00~18:59) on Halloween; and (b) injuries that occur between 17:00 and 18:00 (17:00~17:59) on Halloween are more severe than those occurring at any other time and day.

A few studies have revealed no significant difference in relative risks during the Halloween season in most situations, including vehicle accidents, accidental poisoning and drowning, and adverse drug effects [13]. Our results derived from the U.K. STATS19 dataset are similar to studies reporting a 49% increase in the risks of pediatric fatal pedestrian crashes on Halloweens in the United States [14,15]. In addition to the results showing a higher number of fatalities on this holiday, our study contributes to the pediatric injury literature by concluding that injury severity is the highest between 17:00 and 18:00 (17:00~17:59) on Halloween. The general rule appears to be trick-or-treating starts around 16:00 or 17:00 and should end around 20:00 [16]. The rush hour is a time when commuters and students are on their way to work/school and, in the United Kingdom, this is generally considered to be between 16:00 and 19:00 in the evening [17]. The activities on the Halloween start during the rush hour, which may lead to more traffic casualties and more severe injuries. Our result indicates that parents, children, and drivers should stay alert to the potential danger of crashes during this time.

Recently, scholars have reported that insufficient awareness of road safety, high degree of urbanization, high population density, and high traffic volume are risk factors for pediatrics casualties. The dusk hours—late afternoon and early evening—are also a high-risk period [15,18]. Some studies have demonstrated that distraction may have an adverse effect on pediatric pedestrian safety [12,19,20,21]. Trick-or-treating is one of main activities conducted on Halloween. Children dress up in various costumes such as a ghost, ring each neighbor’s doorbell, and shout “trick or treat!” Wearing a costume at night and knocking on doors increase the risk of a crash.

According to the report from the European, in the pedestrian casualties, some factors such as male, crash with the car, and area speed limit of about 30 mph are associated with severe injuries [22]. Our study also has similar results.

The World Health Organization has proposed the 3E strategies—education, engineering, and enforcement—for reducing the risks to pedestrian children [23]. A comprehensive model should be developed and should combine education with community and environmental interventions. The public and parents must recognize that children participating in various activities on Halloween have a high risk of being involved in an accident and being severely injured. Strengthening safety education for both parents and children—adding reflective panels to costumes, increasing numbers of community road caretakers, and reminding drivers to slow down on this holiday—may be effective countermeasures.

## 5. Limitations

This study has some limitations. First, limited data were available for some independent variables, such as the education level of parents, community economy, traffic volume, and geographic distribution of communities and road types, and these variables may crucially affect the number and type of crashes involving children. Moreover, this study used a national database in which information on injury sites was unavailable. Analysis with consideration of different injury sites is necessary for developing effective preventive policies. Further studies should include these factors and a dataset combined with hospital-based data in analysis. In addition, many factors are insignificant, which may be affected by the heterogeneity of individuals; a mixed logit model such as random parameters Bayesian LASSO modeling may be used in the future study to resolve the problem [24].

## 6. Conclusions

Children are more likely to sustain road traffic injuries on this holiday. Our research indicates that, between 17:00 and 19:00 (17:00~18:59) on Halloween days, the numbers of all injuries and specifically KSIs among pediatrics traffic casualties were higher than those on all the other day types. Moreover, for a crash that occurred between 17:00 and 18:00 (17:00~17:59) on Halloween, KSIs were 34.2% more likely in pediatrics casualties than on other days and at other times. Our results indicate that parents, children, and drivers should stay alert to the potential dangers faced by children during this time.

## Figures and Tables

**Table 1:** Characteristics of casualties from 1990 to 2017.

Characteristic	Number of Casualties	Percentage (%)
Sex		
Female	43,625	59.28
Male	29,962	40.72
Road type		
Rural	6504	8.84
Urban	67,083	91.16
Weather		
Fine	59,076	80.28
Adverse	14,511	19.72
Road condition		
Dry	46,753	63.53
Not dry	26,834	36.47
Period of 16:00 to 00:00		
Weekday	56,041	71.16
Weekend day	831	1.13
Public holiday	15,308	20.80
Halloween	1407	1.91
Period of 16:00 to 17:00		
Weekday	8508	85.27
Weekend day	59	0.59
Public holiday	1283	12.86
Halloween	128	1.28
Period of 17:00 to 18:00		
Weekday	9645	79.56
Weekend day	43	0.35
Public holiday	2062	17.01
Halloween	373	3.08
Period of 18:00 to 19:00		
Weekday	10,284	77.90
Weekend day	49	0.37
Public holiday	2500	18.94
Halloween	368	2.79
Period of 19:00 to 20:00		
Weekday	9679	75.81
Weekend day	160	1.25
Public holiday	2670	20.91
Halloween	259	2.03
Period of 20:00 to 21:00		
Weekday	7269	71.58
Weekend day	233	2.29
Public holiday	2509	24.71
Halloween	144	1.42
Period of 21:00 to 22:00		
Weekday	5828	72.45
Weekend day	153	1.90
Public holiday	1995	24.80
Halloween	68	0.85
Period of 22:00 to 23:00		
Weekday	3073	68.36
Weekend day	78	1.74
Public holiday	1298	28.88
Halloween	46	1.02
Period of 23:00 to 00:00		
Weekday	1755	62.17
Weekend day	56	1.98
Public holiday	991	35.10
Halloween	21	0.74
Type of vehicle involved		
Automobile	66,926	90.95
Large vehicle	4215	5.73
Motorcycle	2105	2.86
Bicycle	341	0.46
Injury severity		
Slight injury	55,358	75.23
KSI	18,229	24.77
Total	73,587	100.00

**Table 2:** Distribution of injury severity by risk factor during 1990 to 2017.

Characteristics	KSI n (%)	Slight Injury n (%)	*p*-Value
Sex			<0.001
Female	6943 (23.17%)	23,019 (76.83%)	
Male	11,286 (25.87%)	32,339 (74.13%)	
Road type			<0.001
Rural	2649 (40.73%)	3855 (59.27%)	
Urban	15,580 (23.22%)	51,503 (76.78%)	
Weather			<0.001
Fine	14,912 (25.24%)	44,164 (74.76%)	
Adverse	3317 (22.86%)	11,194 (77.14%)	
Road condition			0.662
Dry	11,557 (24.72%)	35,196 (75.28%)	
Not dry	6672 (24.86%)	20,162 (75.14%)	
Period of 16:00 to 17:00			0.450
Weekday	1865 (21.92%)	6643 (78.08%)	
Weekend day	17 (28.81%)	42 (71.19%)	
Public holiday	296 (23.07%)	987 (76.93%)	
Halloween	26 (20.31%)	102 (79.69%)	
Period of 17:00 to 18:00			0.021
Weekday	2267 (23.50%)	7378 (76.50%)	
Weekend day	8 (18.60%)	35 (81.40%)	
Public holiday	527 (25.56%)	1535 (74.44%)	
Halloween	108 (28.95%)	265 (71.05%)	
Period of 18:00 to 19:00			0.304
Weekday	2487 (24.18%)	7797 (75.82%)	
Weekend day	13 (26.53%)	36 (73.47%)	
Public holiday	649 (25.96%)	1851 (74.04%)	
Halloween	93 (25.27%)	275 (74.73%)	
Period of 19:00 to 20:00			0.576
Weekday	2490 (25.73%)	7189 (74.27%)	
Weekend day	34 (21.25%)	126 (78.75%)	
Public holiday	699 (26.18%)	1971 (73.82%)	
Halloween	68 (26.25%)	191 (73.75%)	
Period of 20:00 to 21:00			0.705
Weekday	1825 (25.11%)	5444 (74.89%)	
Weekend day	54 (23.18%)	179 (76.82%)	
Public holiday	642 (25.59%)	1867 (74.41%)	
Halloween	32 (22.22%)	112 (77.78%)	
Period of 21:00 to 22:00			0.040
Weekday	1404 (24.09%)	4424 (75.91%)	
Weekend day	38 (24.84%)	115 (75.16%)	
Public holiday	544 (27.27%)	1451 (72.73%)	
Halloween	15 (22.06%)	53 (77.94%)	
Period of 22:00 to 23:00			0.342
Weekday	851 (27.69%)	2222 (72.31%)	
Weekend day	16 (20.51%)	62 (79.49%)	
Public holiday	367 (28.27%)	931 (71.73%)	
Halloween	16 (34.78%)	30 (65.22%)	
Period of 23:00 to 00:00			0.903
Weekday	486 (27.69%)	1269 (72.31)	
Weekend day	14 (25.00%)	42 (75.00%)	
Public holiday	271 (27.35%)	720 (72.65%)	
Halloween	7 (33.33%)	14 (66.67%)	
Type of vehicle involved			<0.001
Automobile	16,383 (24.48%)	50,543 (75.52%)	
Large vehicle	1292 (30.65%)	2933 (69.35%)	
Motorcycle	477 (22.66%)	1628 (77.34%)	
Bicycle	77 (22.58%)	264 (77.42%)	

**Table 3:** Logistic regression results of factors associated with KSI casualties.

Variables	β	Standard Error	AOR (95% CI)	*p*-Value
Sex				
Male	0.145	0.018	1.156 (1.117–1.197)	<0.001
Female (ref)	-			
Road type				
Rural	0.816	0.027	2.261 (2.144–2.385)	<0.001
Urban (ref)	-			
Weather				
Fine	0.190	0.027	1.209 (1.148–1.274)	<0.001
Adverse (ref)	-			
Road condition				
Not dry	0.092	0.022	1.100 (1.051–1.143)	<0.001
Dry (ref)	-			
Type of vehicle involved				
Motorcycle	−0.027	0.141	0.974 (0.739–1.283)	0.850
Automobile	0.367	0.135	1.443 (1.108–1.879)	0.006
Large vehicle	0.065	0.131	1.067 (0.826–1.378)	0.621
Bicycle (ref)	-			
Period of 16:00 to 17:00				
Halloween	−0.032	0.222	0.969 (0.626–1.498)	0.886
Public holiday	0.092	0.072	1.096 (0.952–1.262)	0.203
Weekend day	0.406	0.291	1.501 (0.849–2.652)	0.162
Weekday (ref)	-		-	
Period of 17:00 to 18:00				
Halloween	0.294	0.118	1.342 (1.065–1.692)	0.013
Public holiday	−0.108	0.056	1.114 (0.997–1.244)	0.057
Weekend day	−0.356	0.396	0.701 (0.322–1.524)	0.369
Weekday (ref)	-		-	
Period of 18:00 to 19:00				
Halloween	0.078	0.123	1.081 (0.849–1.376)	0.527
Public holiday	0.095	0.052	1.100 (0.994–1.216)	0.066
Weekend day	0.119	0.327	1.127 (0.593–2.140)	0.716
Weekday (ref)	-		-	
Period of 19:00 to 20:00				
Halloween	0.053	0.144	1.054 (0.795–1.398)	0.714
Public holiday	0.026	0.050	1.027 (0.931–1.133)	0.599
Weekend day	−0.199	0.195	0.820 (0.559–1.202)	0.309
Weekday (ref)	-		-	
Period of 20:00 to 21:00				
Halloween	−0.159	0.204	0.853 (0.572–1.271)	0.434
Public holiday	0.009	0.054	1.010 (0.908–1.121)	0.867
Weekend day	−0.125	0.159	0.883 (0.647–1.206)	0.433
Weekday (ref)	-		-	
Period of 21:00 to 22:00				
Halloween	−0.125	0.296	0.882 (0.493–1.577)	0.672
Public holiday	0.156	0.060	1.170 (1.040–1.314)	0.009
Weekend day	0.071	0.191	1.073 (0.738–1.560)	0.712
Weekday (ref)	-		-	
Period of 22:00 to 23:00				
Halloween	0.212	0.319	1.237 (0.662–2.311)	0.506
Public holiday	0.013	0.075	1.013 (0.875–1.173)	0.862
Weekend day	−0.389	0.286	0.678 (0.387–1.188)	0.174
Weekday (ref)	-		-	
Period of 23:00 to 00:00				
Halloween	0.257	0.474	1.293 (0.511–3.272)	0.588
Public holiday	−0.023	0.091	0.977 (0.818–1.168)	0.799
Weekend day	−0.054	0.320	0.947 (0.506–1.773)	0.865
Weekday (ref)	-		-	

## Data Availability

The current research used the U.K. STATS19 database, which contains the data of all road traffic accidents.
